# The influence of the superimposition procedure and type of intraoral impression on the superimposition accuracy of CBCT scans with dental impressions in implant planning: an in-vitro study

**DOI:** 10.1186/s40729-025-00612-y

**Published:** 2025-03-28

**Authors:** Constantin Motel, Carolin Kirschner, Felix Förtsch, Mayte Buchbender, Manfred Wichmann, Ragai-Edward Matta

**Affiliations:** 1 Dental Clinic 2 – Department of Prosthodontics, Erlangen University Hospital, Glückst 11, 91054 Erlangen, Germany; 2 Department of Oral- and Maxillofacial Surgery, Erlangen University Hospital, Glückst 11, 91054 Erlangen, Germany

**Keywords:** Cone beam computer tomography (CBCT), Digital impression, Conventional impression, Digital workflow, Implantology, Superimposition procedure, Implant planning

## Abstract

**Purpose:**

The aim of this study was to investigate the superimposition of CBCT data with virtual models of the oral situation directly generated using an IOS and with indirectly generated plaster models.

**Methods:**

Two different radiopaque jaw models were first scanned using a CBCT unit. Secondly, ten scans using an IOS and ten alginate impressions were made. The alginate impressions were cast with plaster and the plaster models were digitized using a laboratory scanner. Virtual Reference models generated with an industrial scanner were superimposed with the data sets of the virtual models using both a best-fit procedure on the palate and on the teeth. Deviations in two toothless areas were statistically evaluated.

**Results:**

The superimposition of the directly generated models with the CBCT-based datasets showed lower deviations. Lower deviations were also calculated for the best-fit based on the teeth. The lowest deviations were found for model 1 with direct modeling and superimposition over the teeth with 0.008 mm (indirect: 0.210 mm; P = 0.001) and for model 2 with 0.010 mm (indirect 0.106 mm; P = 0.002).

**Conclusions:**

Virtual models of the oral situation generated directly using an IOS are better suited for superimposing with CBCT-based datasets than indirectly generated ones. The best-fit on the teeth is superior to that on the palate.

**Graphical Abstract:**

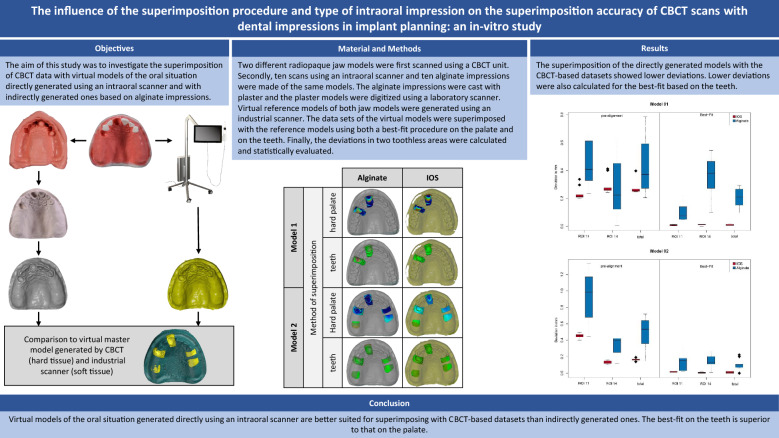

## Introduction

Treatment with osseointegrated dental implants after tooth loss has been a recognized and well-studied treatment option for several decades [1]. The options range from the replacement of individual teeth to the complete rehabilitation of edentulous jaws. Both fixed and removable restorations are possible and offer a wide range of variations that can be selected and modified according to the specific patient situation [2–4]. Once the indication has been determined, the treatment process is divided into imaging and the subsequent planning of the number and positioning of the implants to be inserted as well as the subsequent implant impression, restoration planning and restoration fabrication [5]. This workflow was established over decades and was based on conventional work steps, such as two-dimensional and later three-dimensional imaging, manual fabrication of surgical guides and physical implant impressions as well as the conventional fabrication of implant-supported prosthetics using the lost mold technique [6, 7]. In the course of digitalization, a fundamental change in working methods is also taking place in medicine in general and in dentistry and dental implantology in particular. All sequences of the implantology workflow can now be carried out with computer support (digital workflow) [8–10]. CAD/CAM (computer aided design/computer aided manufacturing) based procedures are already more widely used than conventional techniques for the planning and fabrication of implant-supported dental prostheses [11]. In the field of implant impressions, there is also a progressive development towards the use of digital intraoral scanners, which enables the direct transfer of the oral situation and its availability for planning the superstructure [12, 13]. The new digital technologies also enable procedures that can facilitate the clinical workflow with regard to planning as the first step of implant positioning, which has a strong influence on further treatment. Cone beam computer tomography can be used to image the bony, oral structures. This is a widely accepted and established procedure and enables the three-dimensional assessment of the potential implant site [14, 15]. For three-dimensional planning of implant positioning, highly accurate information about the soft tissue conditions is also of great importance and can also be decisive for the esthetic outcome and patient satisfaction [16–18]. Three-dimensional data, in particular on the quantitative composition of the gingiva, can be superimposed in digital form with Cone Beam Computertomography (CBCT) data to create a virtual model of hard and soft tissue. This information plays an important role in implant surgery and implant-prosthetic therapy [19]. For this purpose, on the one hand, conventionally created situation models, for example based on alginate impressions and subsequent fabrication of plaster models, can be digitized using a laboratory scanner. On the other hand, direct digital intraoral impressions can be made to obtain a virtual, digital model of the oral situation [20]. Here, the accuracy of the virtual model and its overlay with the corresponding CBCT data set is of great importance. As far as the authors are aware, there is currently no scientific data available on the factors influencing the superimposition accuracy. It can be assumed that the impression modality, i.e. whether a direct or indirect impression of the oral situation was taken, has an influence on the superimposition accuracy. In addition, the superimposition procedure could influence the quality of the superimposition. A distinction can be made between the reference areas in the context of the superimposition. Reference regions in the oral cavity, mucosal areas (palate) or teeth can be defined. The procedural difference here could consist in particular in the varying degrees of geometric landmarks. For example, the more prominent shapes of teeth could have a positive effect on the accuracy of the overlay. The aim of this study was to investigate the influence of the impression variant and the superimposition procedure on the quality of the superimposition process. The first null hypothesis of this study was that the accuracy of the overlay of oral structures on CBCT datasets is independent of whether a direct or indirect impression was taken. The second null hypothesis was that the superimposition procedure has no influence on the accuracy of the superimposition.

## Material and methods

**Fig. 1:**
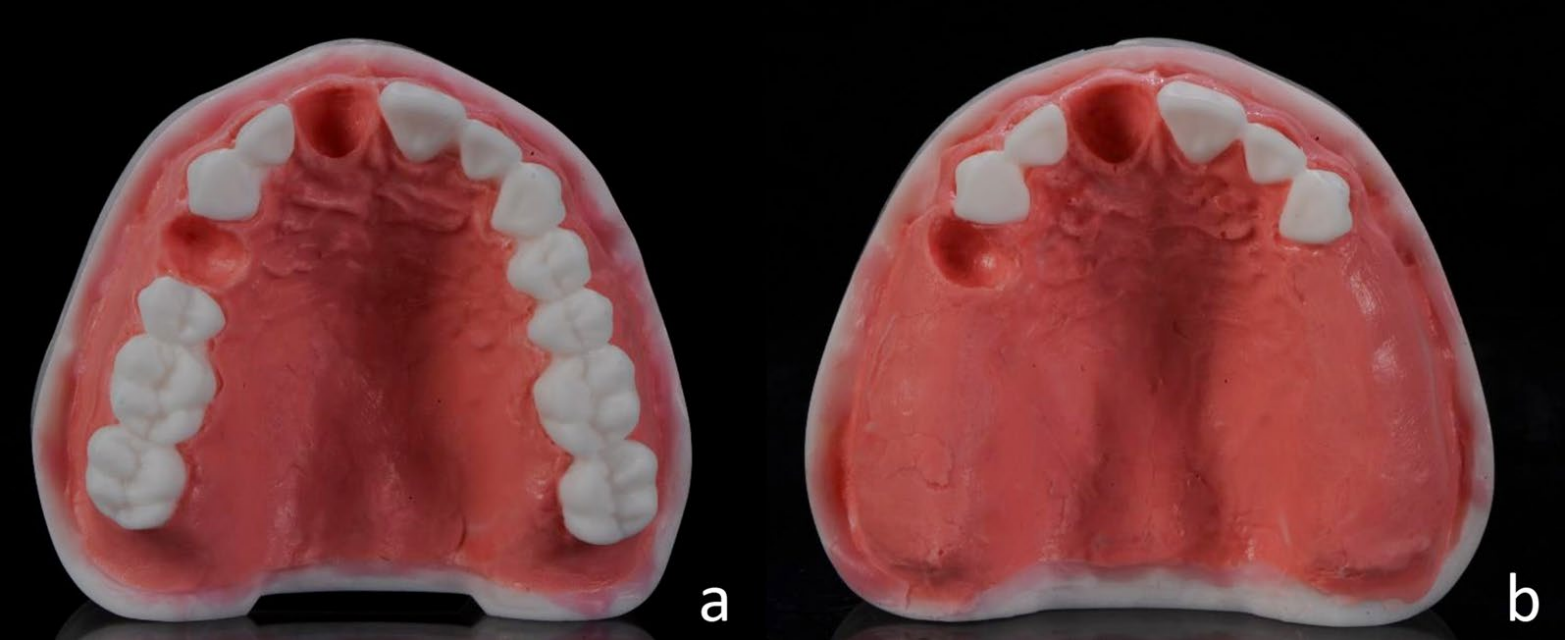
The physical models. Depiction of Models 01 (a) and 02 (b)

The present study was based on two different artificial maxillary models (Model 01 and Model 02). These models were made of radiopaque acrylic to ensure clear radiographic visualization and were toothless in regions 11 and 14. Model 01 was otherwise fully toothed from 17 to 27, while Model 02 had teeth 17–15 and 24–27 removed. Individual gingival masks made of radiopaque silicone were fabricated and attached to the models with cyanoacrylate adhesive. The edges to the model were smoothed by applying pink plate wax. The models are shown in Fig. 1.

The hard tissue representation of the master models was based on CBCT images taken at the Department of Oral and Maxillofacial Surgery at Erlangen University Hospital. The CBCT unit used, ProMax 3D Max (Planmeca, Helsinki, Finland), worked with a flat panel detector. The parameter settings were 0.2 mm, 7.1 mAs, 96 kV and the exposure time was defined as 12 s. The data sets were saved in DICOM format and converted into STL data sets using Impactview 4.4.1 software (CT Imaging GmbH, Erlangen, Germany). Due to the insufficient radiopacity of the gingival mask, an additional optical measurement using a blue light industrial scanner (ATOS II Triple Scan, Carl Zeiss GOM Metrology GmbH, Braunschweig, Germany) was necessary. Reference points were glued to the models and sprayed with a powder-water mixture to reduce light reflections. The scans were loaded as virtual models into the ATOS Professional software (V7.5 SR2, Carl Zeiss GOM Metrology GmbH, Braunschweig, Germany). The scanning accuracy was 4 µm.

**Fig. 2:**
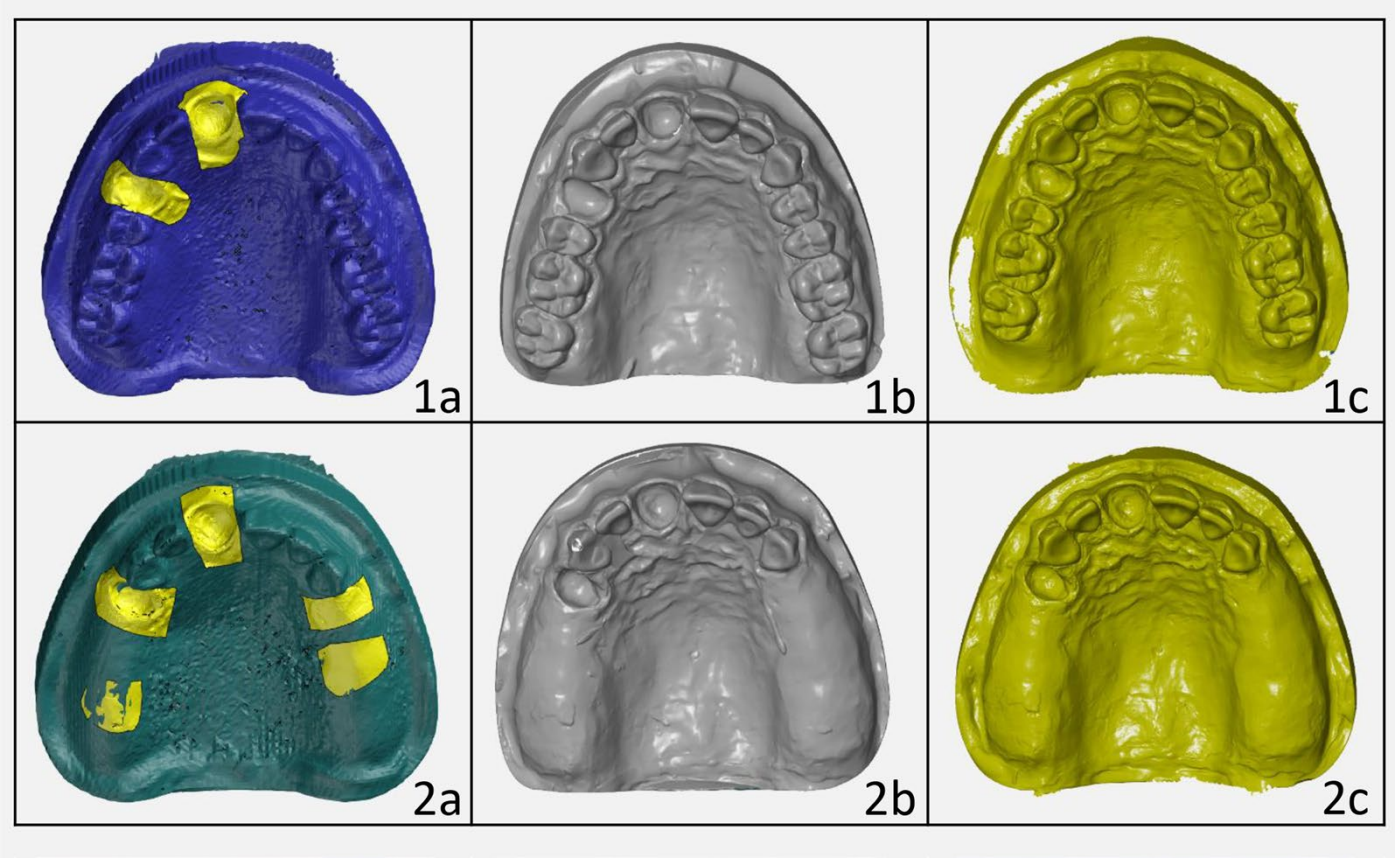
The virtual models. Depiction of the virtual models. 1: Model 01, 2: Model 02. **a** virtual master model, **b** exemplary data set of the intraoral scanner, **c** digitized alginate impression

The Trios 4 intraoral scanner (3Shape A/S, Copenhagen, Denmark) was used for the digital impression. Ten scans each of model 01 and model 02 were taken. The scanning process followed a standardized principle in which the scan head was moved along the dental arches and palate. The scans were checked in real time and saved as STL files. For the conventional impression, ten alginate impressions were made of each model and poured with plaster (Moldano Class 3, Heraeus Kulzer, Hanau, Germany). The alginate used (HS-Maxima® Alginate, Alginate Plus, Henry Schein Dental Deutschland GmbH, Nuremberg, Germany) was dust-free and tear-resistant. The plaster models were digitized in the Erwin H. Schütz dental laboratory (Möhrendorf, Germany) using the Medit T500 laboratory scanner (Medit Corp, Seoul, South Korea). These scans were also saved in open STL format. Figure 2 shows the virtual master models, the directly obtained virtual models using a digital intraoral scanner and the indirectly generated virtual models.

**Fig. 3:**
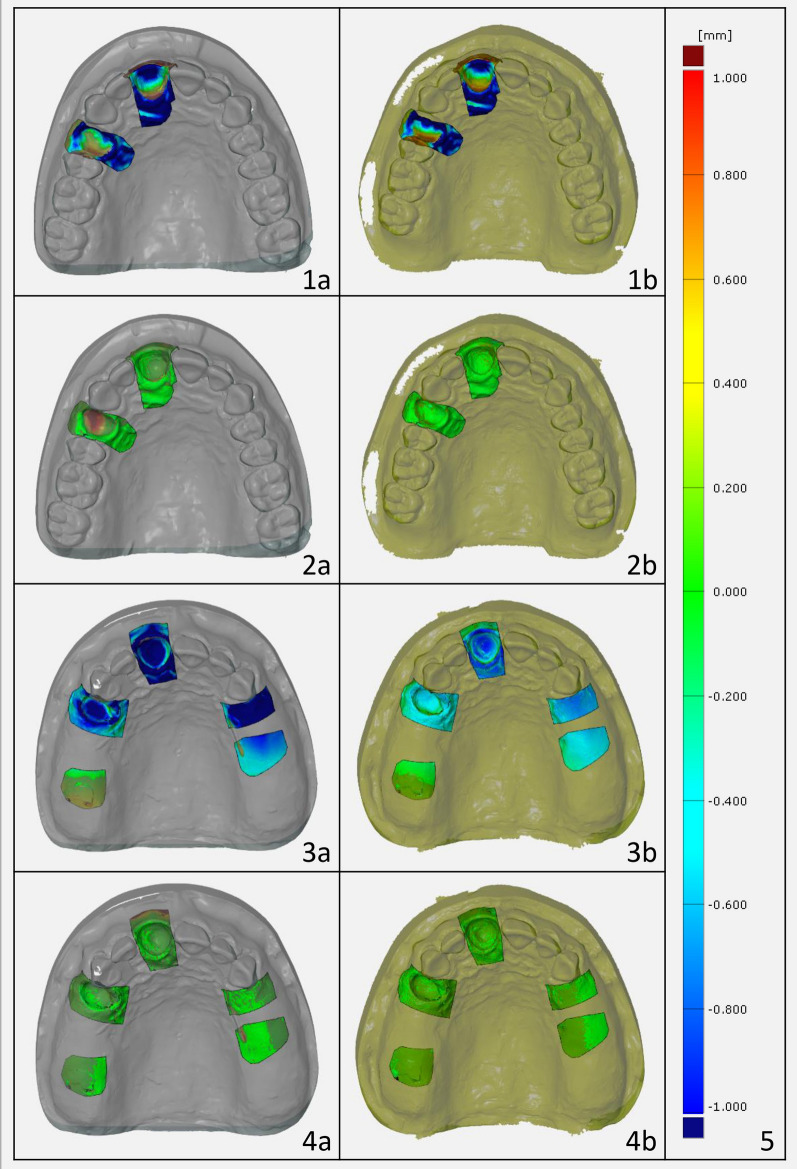
Virtual models after superimposition. Lines 1 and 2: Modell 01, lines 3 and 4: Modell 02. **a** Best-fit on the palate, **b** Best-fit on the teeth

The data sets to be compared were imported individually as STL files into the ATOS Professional software and defined as actual files. The reference models, created from CBCT and ATOS scans, were used as target files for overlaying the impressions. The overlay process consisted of two main steps: Best-fit on the palate: After selecting the reference region on the palate, an algorithm in the software was used for superimposition. Best-fit on the teeth: The best-fit procedure was carried out by making selection of the remaining teeth. In both cases, the reference regions were selected manually in the target file in order to ensure comparability. These procedures were applied to a total of 20 digital impressions and 20 STL data of the laboratory-scanned plaster models (10 per model). Subsequently, the deviations of the digital and conventional impressions from the reference models (master models) were measured and analyzed within regions of interest (ROIs) in the toothless areas 11 and 14 as well as over the entire surface. The ROIs 11 and 14 corresponded to the theoretical implant position in the context of hypothetical implant planning, were marked in the reference models and remained the same for all regarding examinations. In the ATOS Professional software, the perpendicular distance from each point of the actual files to the target file in these ROIs was calculated in millimeters. These deviations were displayed as false color images to visually evaluate the accuracy. Figure 3 shows an example of the overlays using both the best-fit method over the palate and the teeth in the form of the described false color images with corresponding deviation calculations.

The measured values were exported, imported into Microsoft Excel (Microsoft Corporation, Redmond, USA) and finally statistically evaluated using the program R (R Foundation for Statistical Computing, Vienna, Austria). The p-values were calculated using the Mann–Whitney-U test. The significance level was defined as P = 0.05.

## Results

With regard to model 01, the virtual models generated directly with the intraoral scanner showed smaller mean deviations when superimposed with the CBCT data sets than for the indirectly generated virtual models. This applied to both the best-fit on the palate and on the teeth. In the case of the palatal alignment, the smallest mean deviation was 0.232 mm in region 11 for the superimposition of the directly generated virtual model with the CBCT data. The largest mean deviation was calculated for the overlay of the indirectly generated virtual model with the CBCT data, also in region 11, at 0.450 mm. Regarding the best-fit method on the teeth, the smallest mean deviations were recorded in region 11 and over the averaged total area at 0.08 mm for the overlay of the directly generated virtual model with the CBCT data sets. The largest deviation of 0.354 mm was again found in region 14 when overlaying the indirect virtual model with the CBCT data.

**Fig. 4:**
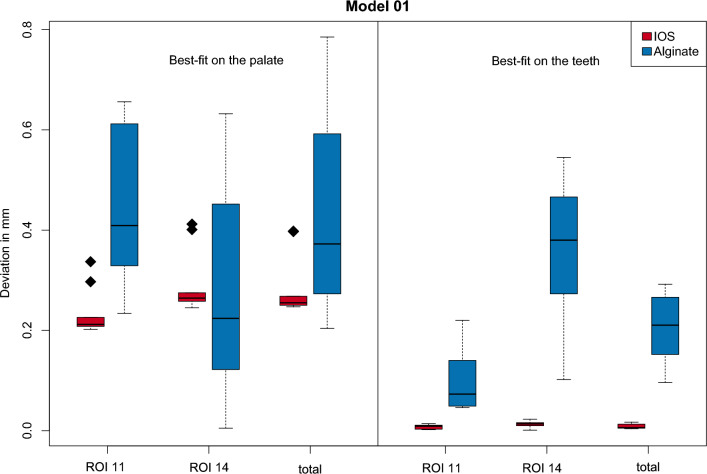
Visualisation of the results (model 01). Box-Whysker plot regarding model 01, comparing best-fit on the palate and best-fit on the teeth concerning regions of interest and underlying data set (IOS vs. Alginate)

**Fig. 5:**
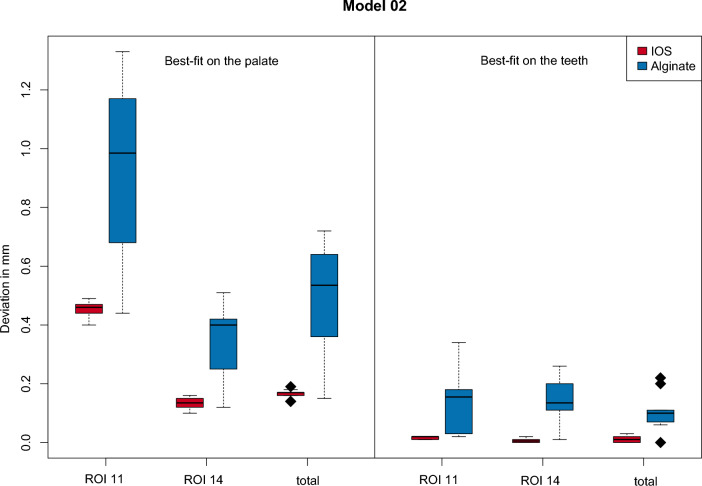
Visualisation of the results (model 02). Box-Whysker plot regarding model 02, comparing best-fit on the palate and best-fit on the teeth concerning regions of interest and underlying data set (IOS vs. Alginate)

With regard to model 02, smaller mean deviations were also calculated for the virtual models generated directly with the intraoral scanner when superimposed with the CBCT data sets, both in the case of the palatal and of the dental alignment. The lowest mean deviation of 0.134 mm was calculated in region 14 for the best-fit based on the palate when the directly generated virtual model was superimposed with the CBCT data set. The largest mean deviation was 0.934 mm in region 11 for the superimposition of the indirectly generated virtual model with the CBCT data. For the best-fit method over the remaining teeth, the smallest mean deviation of 0.006 mm was shown in region 14 when the directly generated virtual model was superimposed with the CBCT data sets. The largest mean deviation was 0.146 mm for the indirectly generated model in region 11. The corresponding descriptive statistics are summarized in Tables 1 and 2. Figure 4 visualizes the data presented for model 01 in the form of a box-whysker plot. The results relating to Model 02 are also shown in Fig. 5 as a box-whysker plot.

**Table 1:** Descriptive statistics for model 01

Procedure	ROI	Virtual model	Mean	SD	Median	Min	Max
Best-fit (palate)	11	Direct	0.232	0.046	0.212	0.202	0.337
Best-fit (palate)	11	Indirect	0.450	0.153	0.409	0.234	0.656
Best-fit (palate)	14	Direct	0.290	0.062	0.264	0.245	0.412
Best-fit (palate)	14	Indirect	0.269	0.207	0.224	0.005	0.632
Best-fit (palate)	Total	Direct	0.283	0.061	0.255	0.247	0.398
Best-fit (palate)	Total	Indirect	0.434	0.206	0.372	0.204	0.785
Best-fit (teeth)	11	Direct	0.008	0.004	0.009	0.002	0.014
Best-fit (teeth)	11	Indirect	0.097	0.060	0.073	0.046	0.220
Best-fit (teeth)	14	Direct	0.013	0.006	0.014	0.001	0.023
Best-fit (teeth)	14	Indirect	0.354	0.160	0.380	0.102	0.545
Best-fit (teeth)	Total	Direct	0.008	0.005	0.006	0.004	0.017
Best-fit (teeth)	Total	Indirect	0.210	0.065	0.211	0.096	0.292
Values in mm

**Table 2:** Descriptive statistics for model 02

Procedure	ROI	Virtual model	Mean1	SD1	Median1	Min1	Max1
Best-fit (palate)	11	Direct	0.452	0.028	0.460	0.40	0.49
Best-fit (palate)	11	Indirect	0.934	0.293	0.985	0.44	1.33
Best-fit (palate)	14	Direct	0.134	0.019	0.135	0.10	0.16
Best-fit (palate)	14	Indirect	0.348	0.127	0.400	0.12	0.51
Best-fit (palate)	Total	Direct	0.165	0.016	0.170	0.14	0.19
Best-fit (palate)	Total	Indirect	0.504	0.180	0.535	0.15	0.72
Best-fit (teeth)	11	Direct	0.016	0.005	0.020	0.01	0.02
Best-fit (teeth)	11	Indirect	0.146	0.117	0.155	0.02	0.34
Best-fit (teeth)	14	Direct	0.006	0.007	0.005	0.00	0.02
Best-fit (teeth)	14	Indirect	0.137	0.076	0.135	0.01	0.26
Best-fit (teeth)	Total	Direct	0.010	0.011	0.010	0.00	0.03
Best-fit (teeth)	Total	Indirect	0.106	0.064	0.100	0.00	0.22
Values in mm

Based on the data presented, it could be shown that the differences in the mean deviations between the directly or indirectly generated virtual models when superimposed with the CBCT data sets were significant in all cases except for model 01 regarding the best-fit procedure on the palate in region 14 and over the averaged total area (P = 0.393 and P = 0.076 respectively). Table 3 shows an overview of the calculated p-values.

**Table 3:** P-values for model 01 and model 02

Model	Procedure	ROI	P-value
Model 01	Best-fit (palate)	11	< 0.001
Model 01	Best-fit (palate)	14	0.393
Model 01	Best-fit (palate)	Total	0.076
Model 01	Best-fit (teeth)	11	< 0.001
Model 01	Best-fit (teeth)	14	< 0.001
Model 01	Best-fit (teeth)	Total	< 0.001
Model 02	Best-fit (palate)	11	0.001
Model 02	Best-fit (palate)	14	0.001
Model 02	Best-fit (palate)	Total	0.002
Model 02	Best-fit (teeth)	11	< 0.001
Model 02	Best-fit (teeth)	14	< 0.001
Model 02	Best-fit (teeth)	Total	0.002

## Discussion

In the presented in-vitro study, the accuracy of the superimposition of data sets of virtual models of the intraoral situation with the data sets of corresponding CBCT images was investigated using two prefabricated jaw models of different dentition. The virtual models were generated directly using a digital intraoral scanner and indirectly by digitizing plaster models based on alginate impressions. Within the ATOS Professional analysis software used, these virtual models were superimposed with the corresponding virtual reference models and the vertical deviations between the data sets were calculated. The principles of this procedure were established by Matta et al and are widely accepted. The methodology was modified by the authors for the study presented. The measurement accuracy was 4 µm [21].

The results of the study presented showed that the virtual situation models generated directly with the digital intraoral scanner led to an overall more accurate overlay with the corresponding CBCT data sets compared to those generated indirectly. In this context, inaccuracies in the impression of the models and their further processing must be taken into account. In the conventional workflow, inaccuracies depending on the impression material used must be calculated in, which in the case of alginate are approx 100 µm regarding to a full arch [22]. The relatively high inaccuracy of this impression material was accepted, as this was the material usually used for situation impressions in everyday clinical practice [23, 24]. On the other hand, there is the so-called plaster error caused by pouring and setting of the plaster. This lies in a range of approx 150 µm [25]. Finally, another possible source of error arises from the digitization of the plaster model using the laboratory scanner. In the case of the scanner used here, the measurement deviation is specified as up to 7 µm. In mathematical terms, this results in a total error range of up to 257 µm when generating the indirect virtual (situation) model, whereby balances of errors could occur in successive work steps. The deviations presented in the study when superimposing the indirectly generated virtual models with the CBCT-based reference models are within this error range in the case of the best-fit procedure, i.e. after superimposition with manually selected references on the remaining teeth in the respective reference model and corresponding scan, with the exception of region 14. The measured deviations after performing the best-fit procedure on the palate are all outside this expected range, which indicates that this method causes a significant procedure-related error. This clearly showed that the superimposition over the remaining teeth had a clear advantage over the procedure based on the denture. This could be explained by the more defined surface structure in the area of the remaining teeth compared to the mucosa areas within the reference models or scans. This results in clear reference points within the superimposition process. Modern digital intraoral scanners, such as the TRIOS 4 used here, achieve average deviations of 45 µm to 120 µm mm in relation to full-arch impressions [26, 27]. Here, the virtual (situation) model is available without any further intermediate step after scanning. By reducing the number of work steps compared to the conventional method, sources of error such as the expansion of plaster or storage-related deformations and subsequent digitization can be eliminated. In addition, digital technology is constantly being developed further, for example by improving the resolution of the scanners and the software performance [27, 28].

With regard to the clinical consequences of the results presented, the error tolerances in the course of the surgical realization of implantological planning must be taken into account. First of all, a safety distance of at least 2 mm from surrounding anatomical structures such as tooth roots, pre-existing implants or nerves in the CBCT is required during surgical implant planning. No comparable mean deviation was shown concerning overlaying either directly or indirectly generated virtual models. In a recent review, it was shown that measurement inaccuracy in the context of low-dose CBCT-based implant planning averages 0.24 mm and is adequate for this purpose [29]. In addition, voxel sizes of 0.3 to 0.4 mm are considered acceptable for implant planning [30]. Based on these values, only the superimpositions of directly generated models with the CBCT-based reference models using the best-fit method after manual selection of the residual teeth consistently achieved tolerable deviations on average. This applies to both Model 01 and Model 02 and indicates that this method could be suitable to support implant planning. In some cases, adequate mean deviations were also shown for the best-fit procedure on the teeth and the indirectly generated virtual models. However, only in the case of model 02 and not for model 01, the superimposition using the best-fit method for indirectly generated models showed consistently acceptable mean deviations. The deviations calculated in the present study, particularly with regard to the best-fit procedure over the residual dentition in direct digital impressions, were significantly smaller than described in the current literature. In a review by Mangano et al., deviations of 0.28 to 0.54 mm were described when superimposing CBCT data sets and scan data from digital intraoral scanners [31]. In addition, the use of resin markers on teeth during CBCT and intraoral scanning is recommended in the current literature to improve the overlay quality of corresponding data sets [32]. Based on the available data from this study, the accuracy of the superimposition with reference to the residual dentition could possibly be sufficient in the future without the use of those markers.

The study presented was subject to various limitations. On the one hand, the deviations were measured when superimposing the indirectly generated models with the corresponding reference models based on alginate impressions, which can exhibit a significantly greater deviation in comparison with impression silicones or modern intraoral scanners in particular and may therefore have influenced the measurements. In addition, this work is the first publication in this field with regard to the superimposition method and must therefore be supplemented by further investigations in the future. Furthermore, additional parameters such as CBCT parameters, characteristics of the oral structures such as edentulism or previous surgery could be included in future studies. The in-vitro character of the present study means that, for example, non-standardized lighting conditions, the absence of saliva and a patient-specific scan duration, as are present in everyday clinical practice, limit the transferability of the study results. In addition, artifacts in CBCT that could not be simulated in the present study are to be expected in vivo. Consequently, in-vivo studies should be carried out to ensure the transfer to clinical practice.

## Conclusions

1. When superimposing virtual models with CBCT-based data sets, models generated directly using intraoral scanners are more suitable than those generated indirectly. The first null hypothesis is rejected.
2. The best-fit method based on manual selection of the residual dentition is superior to the best-fit procedure based on the palate in terms of superimposition accuracy. This could be due to the more strongly defined surface structure of the teeth. The second null hypothesis is rejected.
3. Alginate impressions were only partially suitable as a basis for the digital implantology workflow in this study.
4. Based on the results of this study, it is not necessary to apply resin markers to the teeth for the intraoral scan and/or CBCT imaging when superimposing digital intraoral impressions with CBCT data.

## Data Availability

No datasets were generated or analysed during the current study.

## Abbreviations

CBCT: Cone beam computer tomography
STL: Standard tesselation language
ROI: Region of interest
CAD/CAM: Computer aided design/computer aided manufacturing
DICOM: Digital imaging and communications in medicine

## References

[1] Branemark PI, Adell R, Breine U, Hansson BO, Lindstrom J, Ohlsson A. Intra-osseous anchorage of dental prostheses. I. Experimental studies. Scand J Plast Reconstr Surg. 1969. 10.3109/02844316909036699. 4924041 10.3109/02844316909036699

[2] Lang NP, Pun L, Lau KY, Li KY, Wong MC. A systematic review on survival and success rates of implants placed immediately into fresh extraction sockets after at least 1 year. Clin Oral Implant Res. 2012. 10.1111/j.1600-0501.2011.02372.x. 10.1111/j.1600-0501.2011.02372.x 22211305

[3] Pjetursson BE, Thoma D, Jung R, Zwahlen M, Zembic A. A systematic review of the survival and complication rates of implant-supported fixed dental prostheses (FDPs) after a mean observation period of at least 5 years. Clin Oral Implant Res. 2012. 10.1111/j.1600-0501.2012.02546.x. 10.1111/j.1600-0501.2012.02546.x 23062125

[4] Pieralli S, Kohal RJ, Rabel K, von Stein-Lausnitz M, Vach K, Spies BC. Clinical outcomes of partial and full-arch all-ceramic implant-supported fixed dental prostheses. a systematic review and meta-analysis. Clin Oral Implants Res. 2018. 10.1111/clr.13345. 30306694 10.1111/clr.13345

[5] Spector L. Computer-aided dental implant planning. Dent Clin North Am. 2008. 10.1016/j.cden.2008.05.004. 18805228 10.1016/j.cden.2008.05.004

[6] Keith SE, Miller BH, Woody RD, Higginbottom FL. Marginal discrepancy of screw-retained and cemented metal-ceramic crowns on implants abutments. Int J Oral Maxillofac Implants. 1999;14(3):369–78. 10379110

[7] Vigolo P, Givani A. Clinical evaluation of single-tooth mini-implant restorations: a five-year retrospective study. J Prosthet Dent. 2000. 10.1067/mpr.2000.107674. 10898842 10.1067/mpr.2000.107674

[8] Papaspyridakos P, De Souza A, Bathija A, Kang K, Chochlidakis K. Complete digital workflow for mandibular full-arch implant rehabilitation in 3 appointments. J Prosthodont. 2021. 10.1111/jopr.13356. 33811713 10.1111/jopr.13356

[9] Negreiros WM, Hamilton A, Gallucci GO. A completely digital workflow for the transition from a failed dentition to interim complete-arch fixed implant-supported prostheses: a clinical report. J Prosthet Dent. 2022. 10.1016/j.prosdent.2020.09.037. 33388152 10.1016/j.prosdent.2020.09.037

[10] Blatz MB, Coachman C. The complete digital workflow in implant dentistry. Compend Contin Educ Dent. 2023;44(7):416. 37450680

[11] Blackwell E, Nesbit M, Petridis H. Survey on the use of CAD-CAM technology by UK and Irish dental technicians. Br Dent J. 2017. 10.1038/sj.bdj.2017.407. 28496253 10.1038/sj.bdj.2017.407

[12] Joda T, Gintaute A, Bragger U, Ferrari M, Weber K, Zitzmann NU. Time-efficiency and cost-analysis comparing three digital workflows for treatment with monolithic zirconia implant fixed dental prostheses: a double-blinded RCT. J Dent. 2021. 10.1016/j.jdent.2021.103779. 34391875 10.1016/j.jdent.2021.103779

[13] Marques S, Ribeiro P, Falcao C, Lemos BF, Rios-Carrasco B, Rios-Santos JV, et al. Digital impressions in implant dentistry: a literature review. Int J Environ Res Public Health. 2021. 10.3390/ijerph18031020. 33498902 10.3390/ijerph18031020 PMC7908474

[14] Jacobs R, Salmon B, Codari M, Hassan B, Bornstein MM. Cone beam computed tomography in implant dentistry: recommendations for clinical use. BMC Oral Health. 2018. 10.1186/s12903-018-0523-5. 29764458 10.1186/s12903-018-0523-5 PMC5952365

[15] Al-Humairi A, Ip RHL, Spuur K, Zheng X, Huang B. Visual grading experiments and optimization in CBCT dental implantology imaging: preliminary application of integrated visual grading regression. Radiat Environ Biophys. 2022. 10.1007/s00411-021-00959-x. 34988606 10.1007/s00411-021-00959-x

[16] Grunder U. Stability of the mucosal topography around single-tooth implants and adjacent teeth: 1-year results. Int J Periodontics Restorative Dent. 2000;20(1):11–7. 11203544

[17] Chackartchi T, Romanos GE, Sculean A. Soft tissue-related complications and management around dental implants. Periodontol 2000. 2019. 10.1111/prd.12287. 31407443 10.1111/prd.12287

[18] Schmitt CM, Bruckbauer P, Schlegel KA, Buchbender M, Adler W, Matta RE. Volumetric soft tissue alterations in the early healing phase after peri- implant soft tissue contour augmentation with a porcine collagen matrix versus the autologous connective tissue graft: a controlled clinical trial. J Clin Periodontol. 2021. 10.1111/jcpe.13387. 33047372 10.1111/jcpe.13387

[19] Hammerle CH, Cordaro L, van Assche N, Benic GI, Bornstein M, Gamper F, et al. Digital technologies to support planning, treatment, and fabrication processes and outcome assessments in implant dentistry. Summary and consensus statements. The 4th EAO consensus conference 2015. Clin Oral Implants Res. 2015. 10.1111/clr.12648. 26385624 10.1111/clr.12648

[20] Seelbach P, Brueckel C, Wostmann B. Accuracy of digital and conventional impression techniques and workflow. Clin Oral Investig. 2013. 10.1007/s00784-012-0864-4. 23086333 10.1007/s00784-012-0864-4

[21] Matta RE, Adler W, Wichmann M, Heckmann SM. Accuracy of impression scanning compared with stone casts of implant impressions. J Prosthet Dent. 2017. 10.1016/j.prosdent.2016.07.026. 27881327 10.1016/j.prosdent.2016.07.026

[22] Kong L, Li Y, Liu Z. Digital versus conventional full-arch impressions in linear and 3D accuracy: a systematic review and meta-analysis of in vivo studies. Clin Oral Investig. 2022. 10.1007/s00784-022-04607-6. 35786783 10.1007/s00784-022-04607-6

[23] Ashley M, McCullagh A, Sweet C. Making a good impression: (a “how to” paper on dental alginate). Dent Update. 2005. 10.12968/denu.2005.32.3.169. 15881512 10.12968/denu.2005.32.3.169

[24] Nandini VV, Venkatesh KV, Nair KC. Alginate impressions: a practical perspective. J Conserv Dent. 2008. 10.4103/0972-0707.43416. 20142882 10.4103/0972-0707.43416 PMC2813082

[25] Sharif RA, Abdelaziz KM, Alshahrani NM, Almutairi FS, Alaseri MA, Abouzeid HL, et al. The accuracy of gypsum casts obtained from the disinfected extended-pour alginate impressions through prolonged storage times. BMC Oral Health. 2021. 10.1186/s12903-021-01649-2. 34107952 10.1186/s12903-021-01649-2 PMC8191037

[26] Meneghetti PC, Li J, Borella PS, Mendonca G, Burnett LH Jr. Influence of scanbody design and intraoral scanner on the trueness of complete arch implant digital impressions: an in vitro study. PLoS ONE. 2023. 10.1371/journal.pone.0295790. 38113200 10.1371/journal.pone.0295790 PMC10729975

[27] Schmalzl J, Roth I, Borbely J, Hermann P, Vecsei B. The effect of generation change on the accuracy of full arch digital impressions. BMC Oral Health. 2023. 10.1186/s12903-023-03476-z. 37853398 10.1186/s12903-023-03476-z PMC10585882

[28] Mangano F, Gandolfi A, Luongo G, Logozzo S. Intraoral scanners in dentistry: a review of the current literature. BMC Oral Health. 2017. 10.1186/s12903-017-0442-x. 29233132 10.1186/s12903-017-0442-x PMC5727697

[29] Carneiro ALE, Reis INR, Bitencourt FV, Salgado D, Costa C, Spin-Neto R. Accuracy of linear measurements for implant planning based on low-dose cone beam CT protocols: a systematic review and meta-analysis. Dentomaxillofac Radiol. 2024. 10.1093/dmfr/twae007. 38429951 10.1093/dmfr/twae007 PMC11056743

[30] Fokas G, Vaughn VM, Scarfe WC, Bornstein MM. Accuracy of linear measurements on CBCT images related to presurgical implant treatment planning: a systematic review. Clin Oral Implant Res. 2018. 10.1111/clr.13142. 10.1111/clr.13142 30328204

[31] Mangano C, Luongo F, Migliario M, Mortellaro C, Mangano FG. Combining intraoral scans, cone beam computed tomography and face scans: the virtual patient. J Craniofac Surg. 2018. 10.1097/SCS.0000000000004485. 29698362 10.1097/SCS.0000000000004485

[32] Pedrinaci I, Gallucci GO, Lanis A, Friedland B, Pala K, Hamilton A. Computer-assisted implant planning: a review of data registration techniques. Int J Periodontics Restorative Dent. 2024. 10.11607/prd.7127. 39058947 10.11607/prd.7127

